# Supplementary material for: Vγ1 and Vγ4 gamma-delta T cells play opposing roles in the immunopathology of traumatic brain injury in males
Source: Nat Commun. 2023 Jul 18;14:4286. doi: 10.1038/s41467-023-39857-9 (PMC10354011; doi:10.1038/s41467-023-39857-9)
Supplement: Supplementary file 1 — Supplementary Information [file 41467_2023_39857_MOESM1_ESM.pdf]

Vγ1 and Vγ4 gamma-delta T cells play opposing roles in the immunopathology of traumatic brain injury in males

Hadi Abou-El-Hassan[1#], Rafael M. Rezende[1#], Saef Izzy[1], Galina Gabriely[1], Taha Yahya[1], Bruna K. Tatematsu[1], Karl J. Habashy[1], Juliana R. Lopes[1], Gislane L. V. de Oliveira[1], Amir-Hadi Maghzi[1], Zhuoran Yin[1], Laura M. Cox[1], Rajesh Krishnan[1], Oleg Butovsky[1,2], Howard L. Weiner[1]*

[1]*Ann Romney Center for Neurologic Diseases, Brigham & Women's Hospital, Harvard Medical School, Boston, MA, USA*

[2]*Evergrande Center for Immunologic Diseases, Brigham and Women's Hospital, Harvard Medical School, Boston, MA, USA*

[#]Equally contributing authors

*Corresponding Author:

Howard L. Weiner, MD

E-mail: hweiner@rics.bwh.harvard.edu

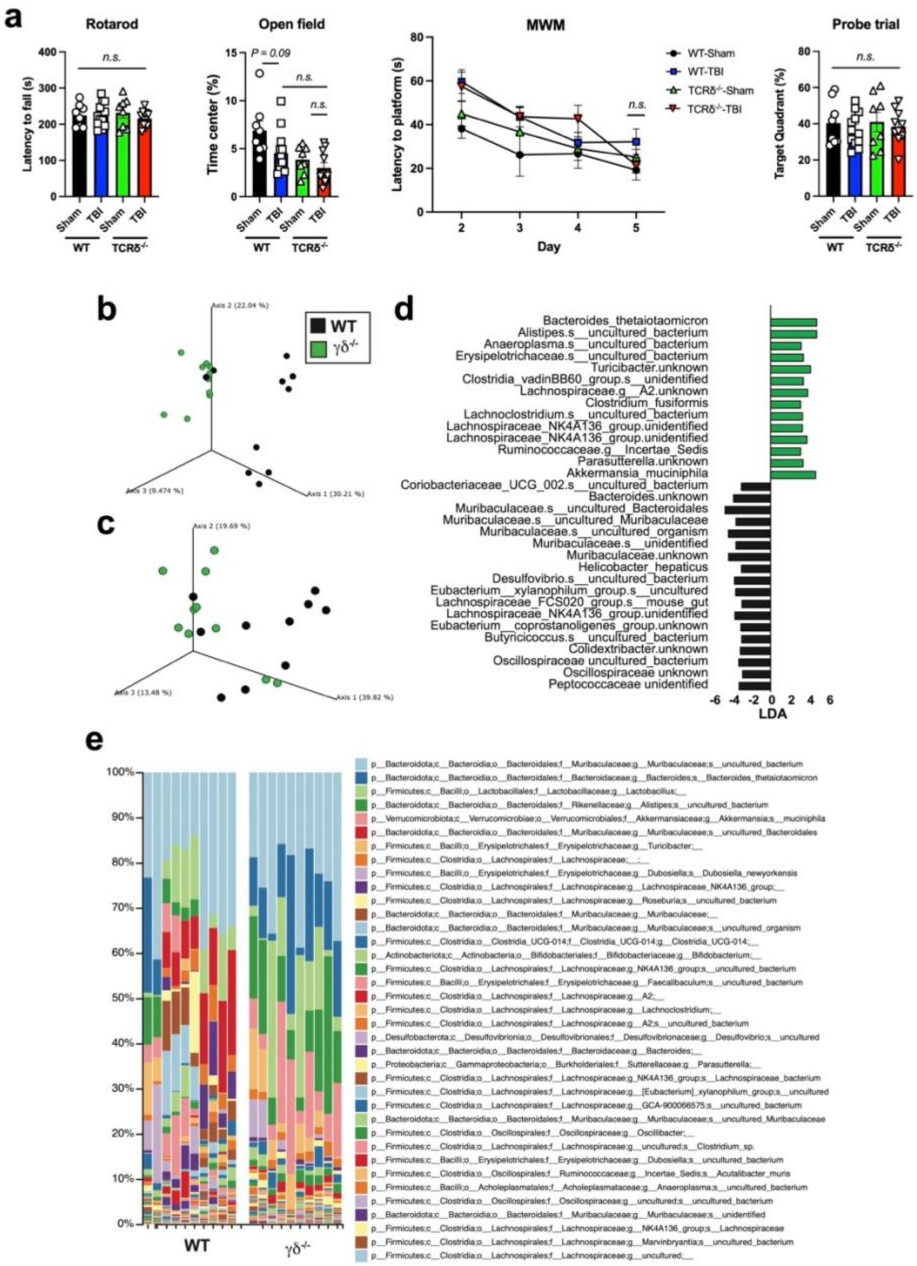

Supplementary Figure 1. Behavioral studies and microbiota composition. (a) Rotarod test, open field test and Morris water maze test in female mice at 1 month after TBI (WT-Sham n = 8, WT-TBI n = 12, TCRδ$^{-/-}$ Sham n = 8, TCRδ$^{-/-}$ TBI n = 10). Biologically independent samples were used. Data shown as mean ± s.e.m. P values calculated by one-way ANOVA with Bonferroni's multiple comparison test for the rotarod, open field and probe trial, and two-way repeated measures ANOVA with Bonferroni's multiple comparison test for the Morris water maze test. **(b-c)** PCoA plot of unweighted **(b)** and weighted **(c)** UniFrac distances of microbiota composition in male WT and TCRδ$^{-/-}$ (γδ$^{-/-}$) mice, PERMANOVA $p < 0.001$. **(d)** Microbiota altered by genotype, reported at the lowest level of identification, effect size plotted, $p < 0.05$ LEfSe. **(e)** Relative abundance of the 37 most prevalent taxa. Source data are provided as a Source Data file.

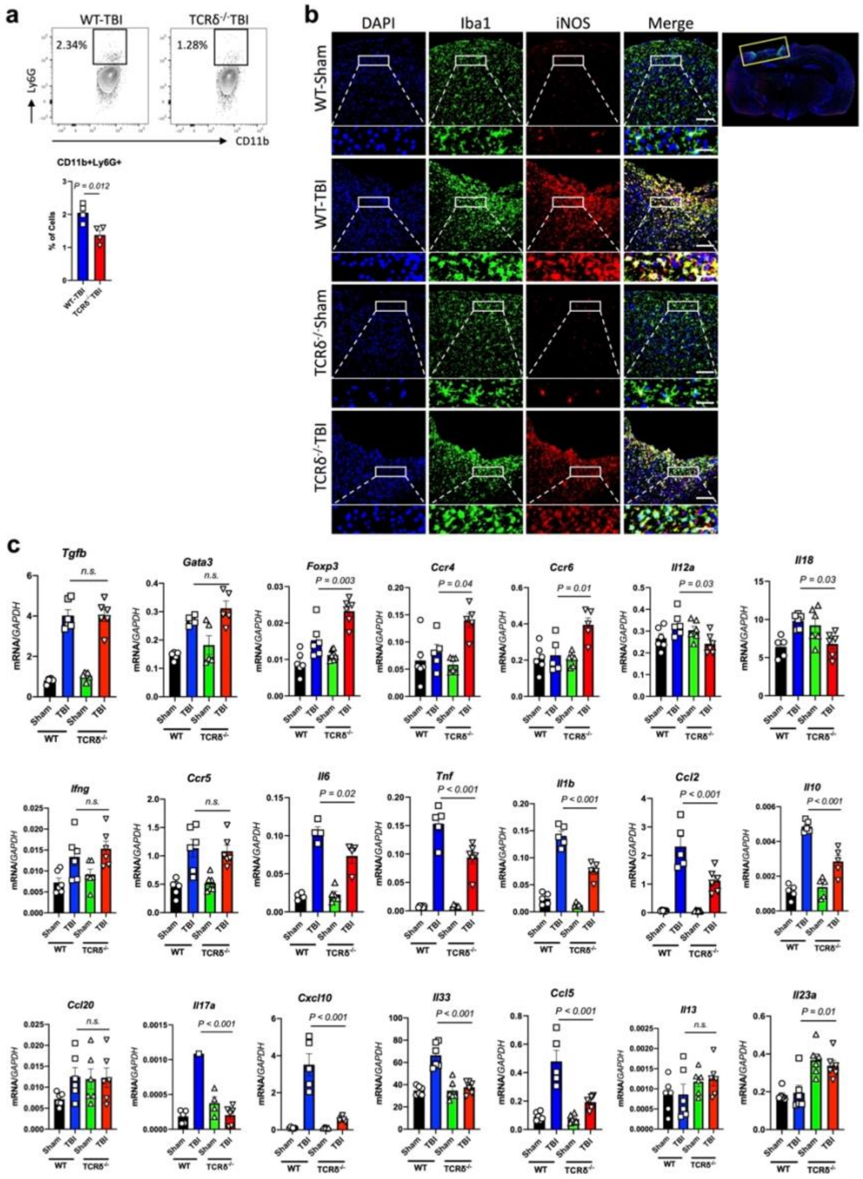

Supplementary Figure 2. Evaluation of neutrophils, immunofluorescence staining and level of expression of cytokines in the pericontusional cortex of TCRδ$^{-/-}$ TBI vs. WT-TBI mice. (a) Flow cytometry analysis and quantification of brain CD11b+Ly6G+ neutrophils (WT-TBI n = 4, TCRδ$^{-/-}$ TBI n = 4). **(b)** Immunofluorescence staining of pericontusional brain samples for DAPI (blue), Iba1 (green) and iNOS (red) along with a merged view of WT-Sham, WT-TBI, TCRδ$^{-/-}$ Sham and TCRδ$^{-/-}$ TBI mice. Dotted box shows a focused area of interest. Scale bars: (upper image) 50 µm, (lower image) 20 µm. **(c)** Quantitative RT-qPCR of cytokines and factors measured from brain lysate of WT-Sham, WT-TBI, TCRδ$^{-/-}$ Sham and TCRδ$^{-/-}$ TBI mice at two days after TBI (n = 1 or 4 or 5 or 6 per group). Biologically independent samples were used. Data shown as mean ± s.e.m. P values calculated by two-tailed unpaired Student's t-test **(a)** and one-way ANOVA with Bonferroni's multiple comparison test **(c)**. Source data are provided as a Source Data file.

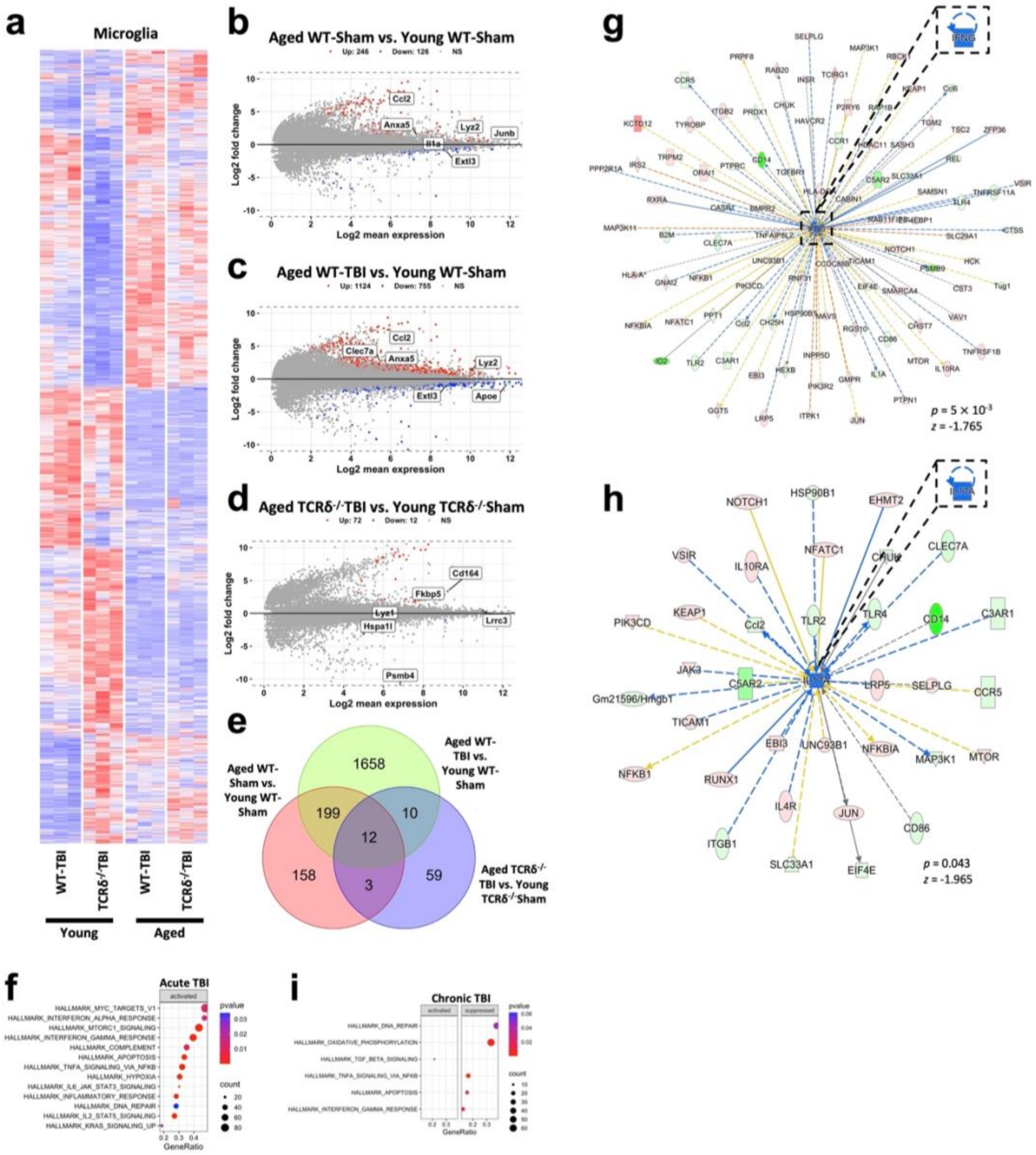

Supplementary Figure 3. γδ T cells modulate microglia in aged mice. (a) Heatmap of the differentially expressed microglia genes mong WT-TBI and TCRδ$^{-/-}$TBI mice, at two days (young) and one year (old) after TBI. **(b-d)** MA plot of the aged WT-Sham vs. young WT-Sham **(b)**, aged WT-TBI vs. young WT-Sham **(c)**, aged TCRδ$^{-/-}$ TBI vs. young TCRδ$^{-/-}$ Sham **(d)** differentially expressed microglia genes. **(e)** Venn diagram of the number of overlapping microglia genes between the three controlled groups. **(f)** GSEA summary plot of acute microglia hallmark pathways. **(g, h)** IPA analysis predicting IFN-γ **(g)** and IL-17A **(h)** as an upstream regulator of the TCRδ$^{-/-}$ TBI vs. WT-TBI acute microglia transcriptome. **(i)** GSEA summary plot of the chronic microglia hallmark pathways. *P* values calculated by FDR-adjusted *P* values with DEseq2 using the Wald test for significance following fitting to a negative binomial linear model and the Benjamini-Hochberg procedure to control for false discoveries **(b, c, d)**, one-tailed t-test **(f, i)** and right-tailed Fisher's exact test **(g, h)**.

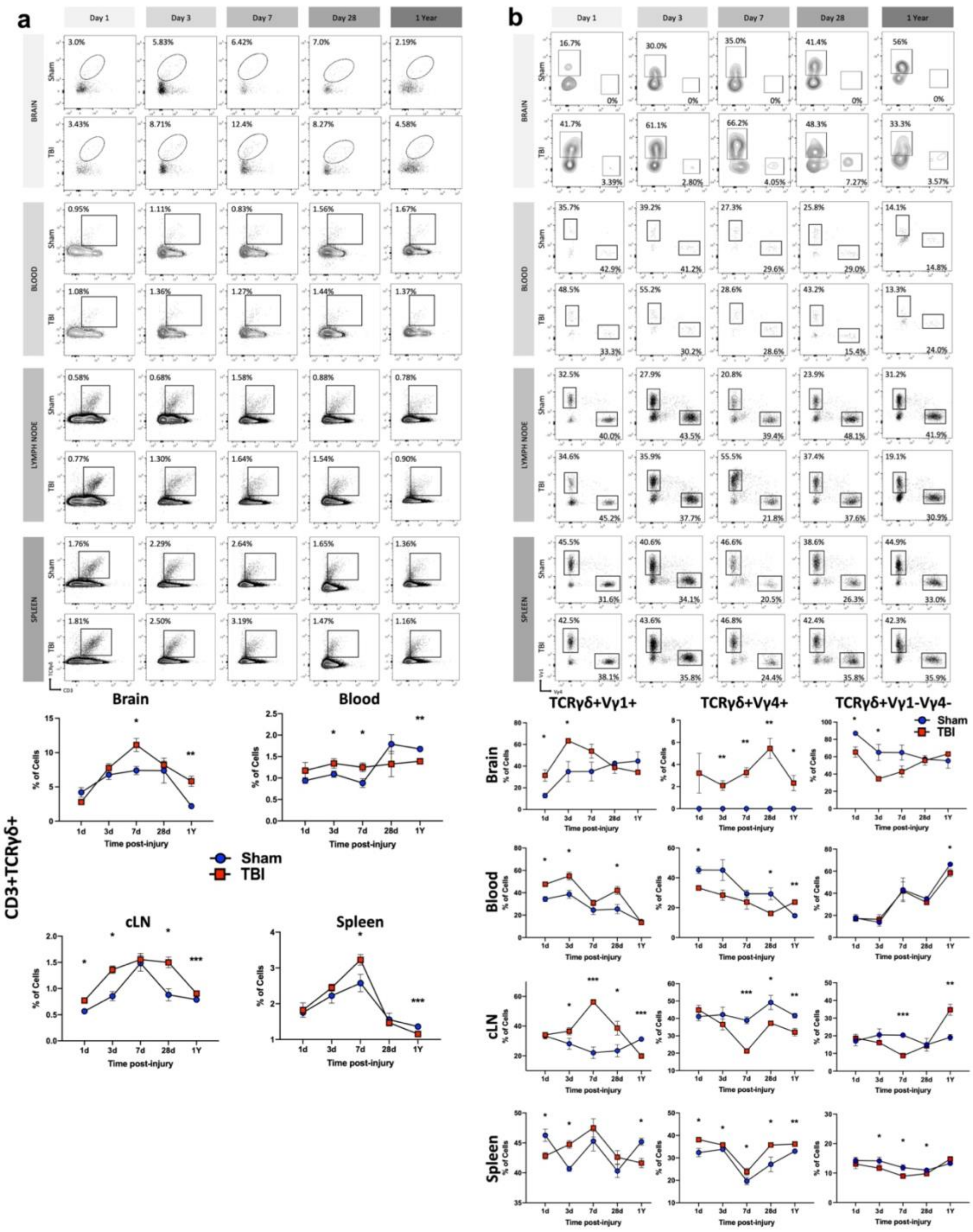

Supplementary Figure 4. Characterization of total and subsets of γδ T cells in TBI. (a, b) Flow cytometry analysis and quantification of total CD3+TCRγδ+ T cells **(a)** and CD3+TCRγδ+Vγ1+, CD3+TCRγδ+Vγ4+ and CD3+TCRγδ+Vγ-Vγ4- subsets **(b)** at one day, three days, one week, one month and one year after TBI in the brain, blood, deep cervical lymph nodes and spleen ($n = 3$ per group). $^{***}P < 0.001$, $^{**}P < 0.01$, $^{*}P < 0.05$. Biologically independent samples were used. Data shown as mean ± s.e.m. P values calculated by two-tailed unpaired Student's t-test. Source data are provided as a Source Data file.

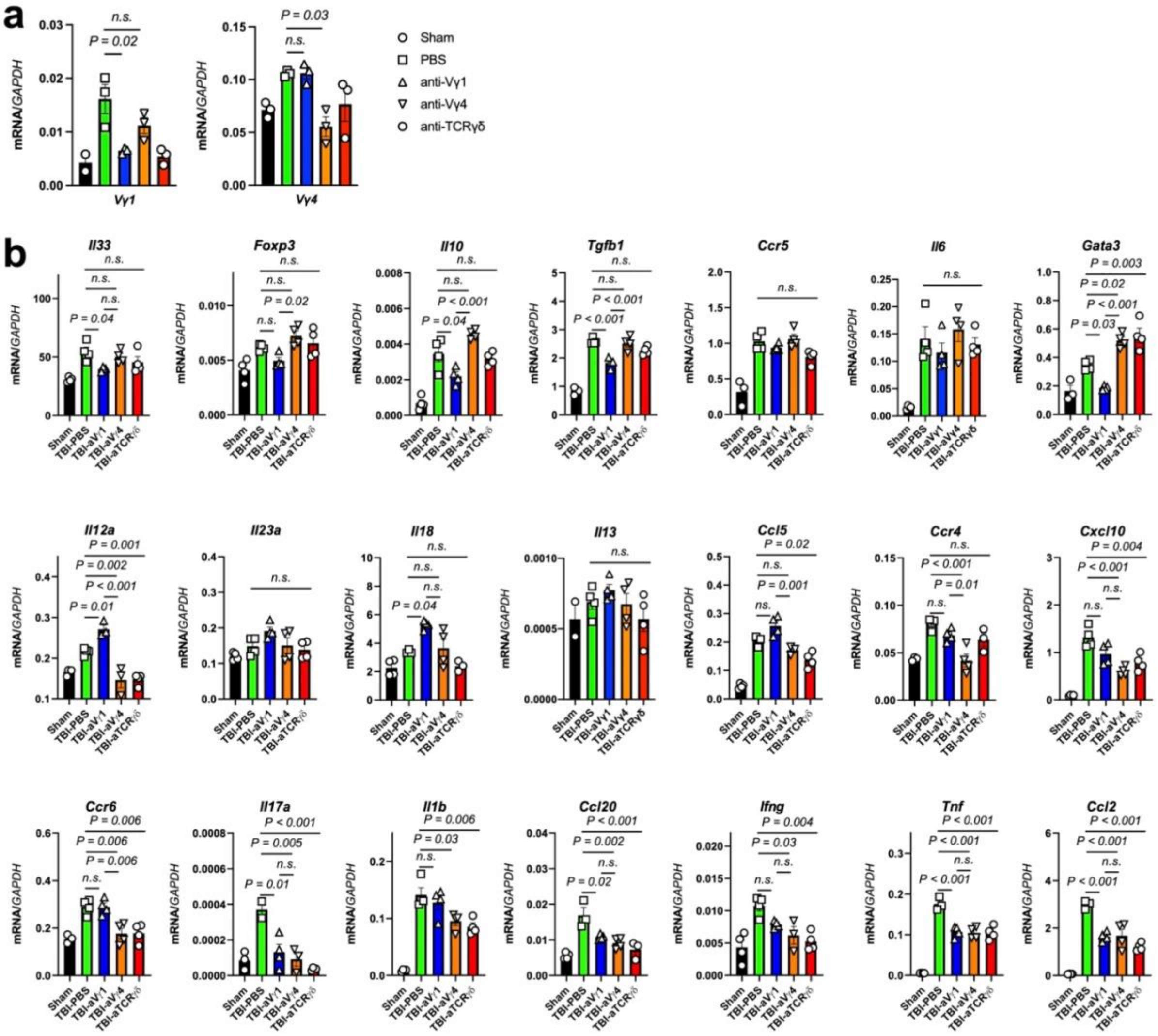

Supplementary Figure 5. Level of expression of cytokines in the pericontusional cortex of wild-type mice after depletion of γδ T cell subsets. (a) Evaluation of the efficacy of γδ T cell depletion by quantitative RT-qPCR of *Vγ1* and *Vγ4* mRNA levels (n = 3 per group). **(b)** Quantitative RT-qPCR of cytokines and factors measured from brain lysate of Sham, TBI-PBS, TBI-aVγ1, TBI-aVγ4 and TBI-aTCRγδ mice at two days after TBI (n = 2 or 3 or 4 per group). Biologically independent samples were used. Data shown as mean ± s.e.m. P values calculated by one-way ANOVA with Bonferroni's multiple comparison test **(a, b)**. aVγ1, anti-Vγ1 depleting antibody; aVγ4, anti-Vγ4 depleting antibody; aTCRγδ, anti-TCRγδ depleting antibody. Source data are provided as a Source Data file.

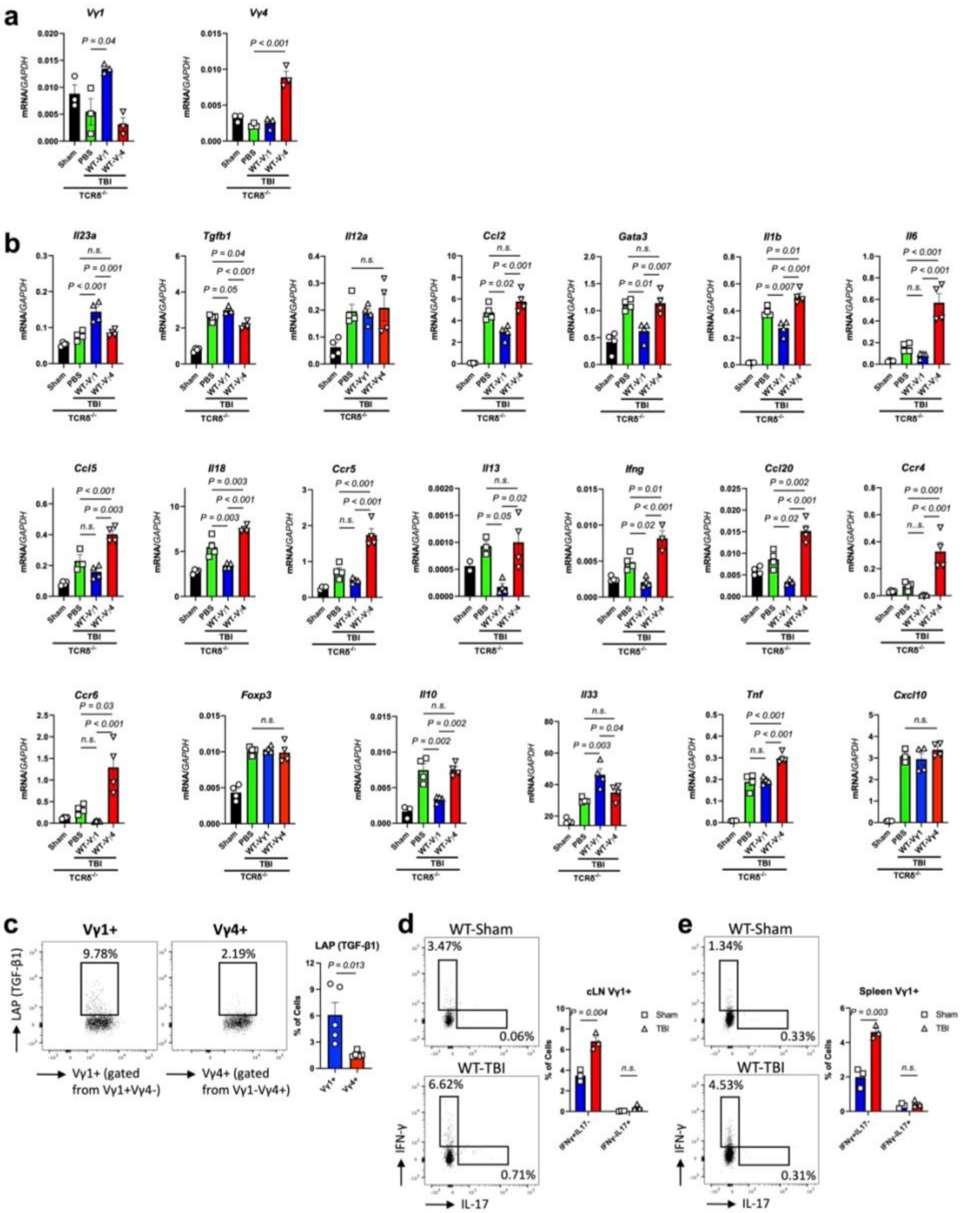

Supplementary Figure 6. Level of expression of cytokines in the pericontusional cortex of TCRδ$^{-/-}$ mice after adoptive transfer of γδ T cell subsets. (a) Evaluation of the efficacy of γδ T cell adoptive transfer by quantitative RT-qPCR of *Vγ1* and *Vγ4* mRNA levels (n = 3 per group). **(b)** Quantitative RT-qPCR of cytokines and factors measured from brain lysate of TCRδ$^{-/-}$ Sham or TBI mice intravenously infused with PBS, WT-Vγ1 or WT-Vγ4 at two days after TBI (n = 2 or 3 or 4 per group). **(c)** Flow cytometry analysis (left) and quantification (right-most) of the expression of LAP (TGF-β1) on Vγ1+Vγ4-, Vγ1-Vγ4+–splenic T cells (n = 5 per group). **(d, e)** Flow cytometry analysis (left) and quantification (right) of intracellular staining of IL-17 and IFN-γ gated from the CD3+TCRγδ+Vγ1+Vγ4- cell population at three days after TBI in the deep cervical lymph nodes **(d)** and spleen **(e)** (n = 3 per group). Biologically independent samples were used. Data shown as mean ± s.e.m. P values calculated by one-way ANOVA with Bonferroni's multiple comparison test **(a, b)** and two-tailed unpaired Student's t-test **(c-e)**. cLN, deep cervical lmph nodes. Source data are provided as a Source Data file.

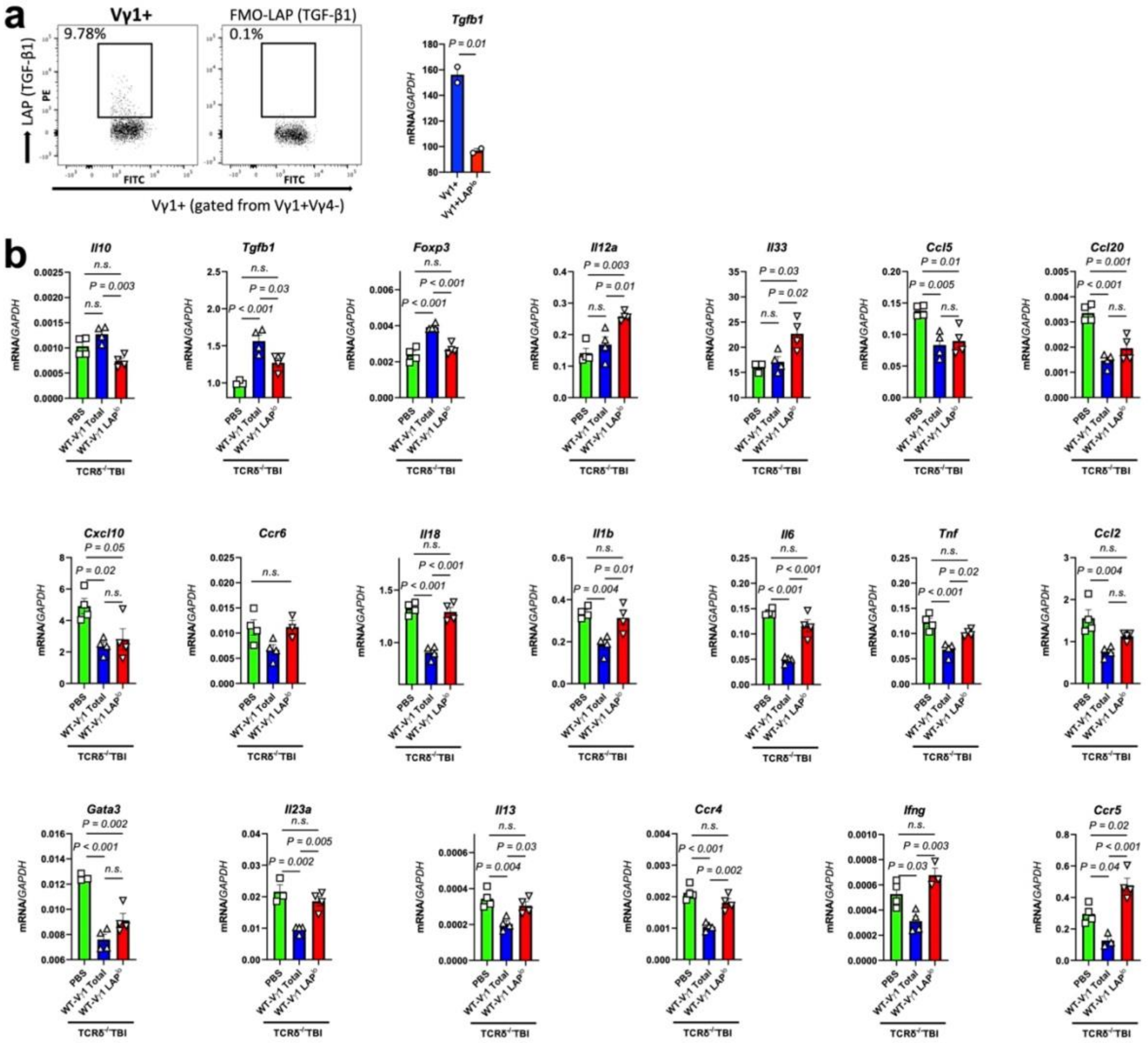

Supplementary Figure 7. Level of expression of cytokines in the pericontusional cortex of TCRδ$^{-/-}$ mice after adoptive transfer of Vγ1 T cells. (a) Validation of the gating strategy (left) via the absence of the regulatory mRNAs in isolated naïve total Vγ1+ and Vγ1+LAP^{lo} splenic T cells by quantitative RT-qPCR of *Tgfb1* mRNA levels (right, n = 3 per group). **(b)** Quantitative RT-qPCR of cytokines and factors measured from brain lysate of TCRδ$^{-/-}$ TBI mice intravenously infused with PBS, WT-Vγ1 or WT-Vγ1LAP^{lo} at two days after TBI (n = 3 or 4 per group). Biologically independent samples were used. Data shown as mean ± s.e.m. P values calculated by two-tailed unpaired Student's t-test **(a)** and one-way ANOVA with Bonferroni's multiple comparison test **(b)**. FMO, fluorescence minus one. Source data are provided as a Source Data file.

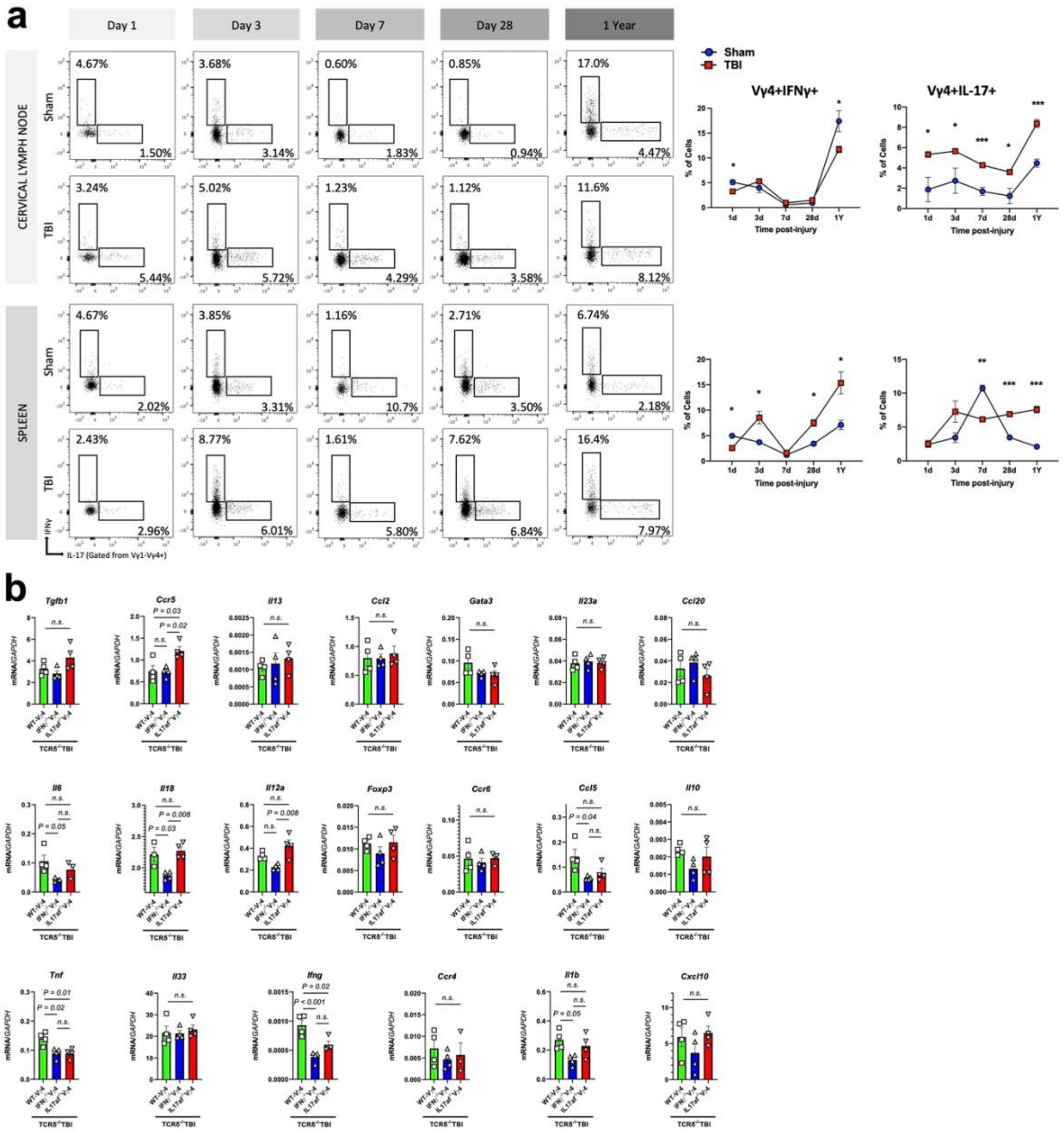

Supplementary Figure 8. Characterization of IL-17 and IFN-γ secreted by γδ T cells in TBI and expression levels of cytokines in the pericontusional cortex of TCRδ$^{-/-}$ mice after adoptive transfer of Vγ4 T cells. (a) Flow cytometry analysis (left) and quantification (right-most) of intracellular staining of IL-17 and IFN-γ gated from the CD3+TCRγδ+Vγ1-Vγ4+ cell population at one day, three days, one week, one month and one year after TBI in the deep cervical lymph nodes and spleen (n = 3 per group). **(b)** Quantitative RT-qPCR of cytokines and factors measured from brain lysate of TCRδ$^{-/-}$ TBI mice intravenously infused with WT-Vγ4, IFNγ$^{-/-}$Vγ4 or IL17af$^{-/-}$Vγ4 at two days after TBI (n = 4 per group). ***P < 0.001, **P < 0.01, *P < 0.05. Biologically independent samples were used. Data shown as mean ± s.e.m. P values calculated by two-tailed unpaired Student's t-test. Source data are provided as a Source Data file.

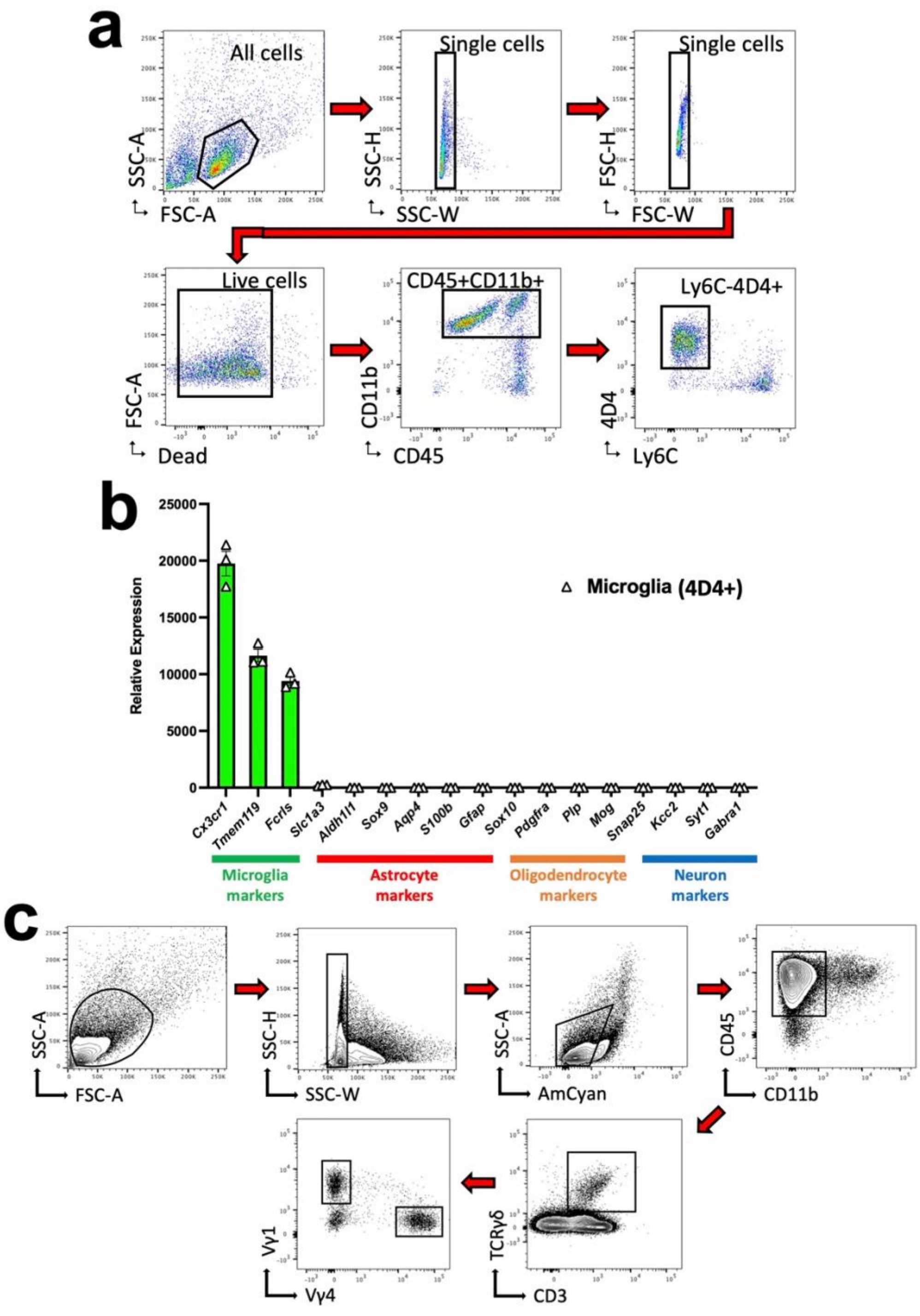

Supplementary Figure 9. Gating strategy for the sorting of microglia single cells and γδ T cells. (a) Flow cytometry dot plots showing the sequences of gates for the isolation of 4D4+ microglia. **(b)** Relative expression of microglia, astrocyte, oligodendrocyte, and neuronal markers by bulk RNAseq in 4D4+ microglia isolated as in **a** from WT mice (*n* = 3 biologically independent samples). **(c)** Flow cytometry dot plots showing the sequences of gates for the isolation of Vγ1 and Vγ4 γδ T cells. Data shown as mean ± s.e.m. Source data are provided as a Source Data file.

a

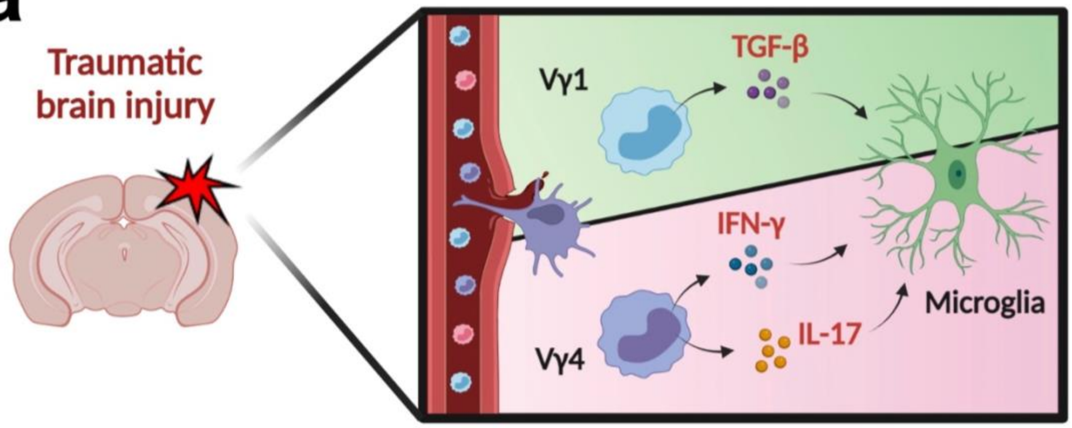

b

Key differentially expressed microglia genes	
**Homeostatic/ Regenerative**	**Inflammatory/ Neurodegenerative**
Tmem119	*Ccl2*
Fkbp5	*Casp1*
Lrrc3	*Tlr4*
Siglech	*Cd86*
Tgfb1	*Hmgb1*
Fcrls	*Stip1*
Atf3	*Il1b*
Nfkbia	*CD74*
Dusp1	*Ccr2*
Egr1	*Ccl5*
Fam49b	*Sod1*
Socs4	*Spp1*
Irf2bpl	*Ccl12*

Supplementary Figure 10. Graphical abstract summarizing the study findings. (a) Vγ1 γδ T cells secrete TGF-β, which promotes a homeostatic microglial profile, whereas Vγ4 γδ T cells secrete IFN-γ and IL-17 resulting in neuroinflammation after TBI. Created with BioRender.com. **(b)** List of key differentially expressed homeostatic/regenerative and inflammatory/neurodegenerative microglia genes.

Supplementary Data 1. List of microglia differentially expressed genes in WT and TCRδ$^{-/-}$ mice by bulk RNAseq. Differential gene expression analysis was performed with an FDR-adjusted *P* values using the Wald test in DESeq2 R package with an adjusted *P* cutoff value of 0.05. *P* values were corrected for multiple testing using the Benjamini-Hochberg method.

Supplementary Data 2. List of microglia differentially expressed genes after depletion of γδ T cell subsets by bulk RNAseq. Differential gene expression analysis was performed with the likelihood ratio test across all groups and FDR-adjusted *P* values using the DESeq2 R package with an adjusted *P* cutoff value of 0.05. *P* values were corrected for multiple testing using the Benjamini-Hochberg method.

Supplementary Data 3. List of microglia differentially expressed genes after adoptive transfer of γδ T cell subsets by bulk RNAseq. Differential gene expression analysis was performed with the likelihood ratio test across all groups and FDR-adjusted *P* values using the DESeq2 R package with an adjusted *P* cutoff value of 0.05. *P* values were corrected for multiple testing using the Benjamini-Hochberg method.

Supplementary Data 4. List of microglia differentially expressed genes after adoptive transfer of Vγ1 T cells by bulk RNAseq. Differential gene expression analysis was performed with an FDR-adjusted *P* values using the Wald test in DESeq2 R package with an adjusted *P* cutoff value of 0.05. *P* values were corrected for multiple testing using the Benjamini-Hochberg method.

Supplementary Data 5. List of microglia differentially expressed genes after adoptive transfer of Vγ4 T cells by bulk RNAseq. Differential gene expression analysis was performed with an FDR-adjusted *P* values using the Wald test in DESeq2 R package with an adjusted *P* cutoff value of 0.05. *P* values were corrected for multiple testing using the Benjamini-Hochberg method.
