# Supplementary material for: Vγ1 and Vγ4 gamma-delta T cells play opposing roles in the immunopathology of traumatic brain injury in males
Source: Nat Commun. 2023 Jul 18;14:4286. doi: 10.1038/s41467-023-39857-9 (PMC10354011; doi:10.1038/s41467-023-39857-9)
Supplement: Supplementary file 3 — Description of Additional Supplementary Files [file 41467_2023_39857_MOESM3_ESM.pdf]

Description of Additional Supplementary Files

Supplementary Data 1. List of microglia differentially expressed genes in WT and TCRδ$^{-/-}$ mice by bulk RNAseq. Differential gene expression analysis was performed with an FDR-adjusted *P* values using the Wald test in DESeq2 R package with an adjusted *P* cutoff value of 0.05. *P* values were corrected for multiple testing using the Benjamini-Hochberg method.

Supplementary Data 2. List of microglia differentially expressed genes after depletion of γδ T cell subsets by bulk RNAseq. Differential gene expression analysis was performed with the likelihood ratio test across all groups and FDR-adjusted *P* values using the DESeq2 R package with an adjusted *P* cutoff value of 0.05. *P* values were corrected for multiple testing using the Benjamini-Hochberg method.

Supplementary Data 3. List of microglia differentially expressed genes after adoptive transfer of γδ T cell subsets by bulk RNAseq. Differential gene expression analysis was performed with the likelihood ratio test across all groups and FDR-adjusted *P* values using the DESeq2 R package with an adjusted *P* cutoff value of 0.05. *P* values were corrected for multiple testing using the Benjamini-Hochberg method.

Supplementary Data 4. List of microglia differentially expressed genes after adoptive transfer of Vγ1 T cells by bulk RNAseq. Differential gene expression analysis was performed with an FDR-adjusted *P* values using the Wald test in DESeq2 R package with an adjusted *P* cutoff value of 0.05. *P* values were corrected for multiple testing using the Benjamini-Hochberg method.

Supplementary Data 5. List of microglia differentially expressed genes after adoptive transfer of Vγ4 T cells by bulk RNAseq. Differential gene expression analysis was performed with an FDR-adjusted *P* values using the Wald test in DESeq2 R package with an adjusted *P* cutoff value of 0.05. *P* values were corrected for multiple testing using the Benjamini-Hochberg method.
