# Supplementary material for: Vγ1 and Vγ4 gamma-delta T cells play opposing roles in the immunopathology of traumatic brain injury in males
Source: Nat Commun. 2023 Jul 18;14:4286. doi: 10.1038/s41467-023-39857-9 (PMC10354011; doi:10.1038/s41467-023-39857-9)
Supplement: Supplementary file 9 — Reporting Summary [file 41467_2023_39857_MOESM9_ESM.pdf]

nature portfolio

Corresponding author(s): Howard L. Weiner

Last updated by author(s): 06/27/2023

Reporting Summary

Nature Portfolio wishes to improve the reproducibility of the work that we publish. This form provides structure for consistency and transparency in reporting. For further information on Nature Portfolio policies, see our Editorial Policies and the Editorial Policy Checklist.

Statistics

For all statistical analyses, confirm that the following items are present in the figure legend, table legend, main text, or Methods section.

n/a	Confirmed	
☐	☒	The exact sample size (*n*) for each experimental group/condition, given as a discrete number and unit of measurement
☐	☒	A statement on whether measurements were taken from distinct samples or whether the same sample was measured repeatedly
☐	☒	The statistical test(s) used AND whether they are one- or two-sided<br>*Only common tests should be described solely by name; describe more complex techniques in the Methods section.*
☐	☒	A description of all covariates tested
☐	☒	A description of any assumptions or corrections, such as tests of normality and adjustment for multiple comparisons
☐	☒	A full description of the statistical parameters including central tendency (e.g. means) or other basic estimates (e.g. regression coefficient) AND variation (e.g. standard deviation) or associated estimates of uncertainty (e.g. confidence intervals)
☐	☒	For null hypothesis testing, the test statistic (e.g. *F*, *t*, *r*) with confidence intervals, effect sizes, degrees of freedom and *P* value noted<br>*Give P values as exact values whenever suitable.*
☐	☒	For Bayesian analysis, information on the choice of priors and Markov chain Monte Carlo settings
☐	☒	For hierarchical and complex designs, identification of the appropriate level for tests and full reporting of outcomes
☒	☐	Estimates of effect sizes (e.g. Cohen's *d*, Pearson's *r*), indicating how they were calculated

Our web collection on statistics for biologists contains articles on many of the points above.

Software and code

Policy information about availability of computer code

Data collection	1.RNA-seq. Samples were processed for cDNA generation and Illumina Nextera XT library construction. Sequencing data was generated using 2x38 bp paired end sequencing on the NextSeq500. 2.Flow cytometry. acquisition was performed on LSRFortessa and FACSSymphony A5 (BD Biosciences) using DIVA software. 3.Immunofluorescence. Images were acquired using a Leica DMi8 widefield microscope. 4.RT-qPCR. Data were collected using a Vii 7 real-time PCR system. 5.Western Blotting. Membranes were exposed on on iBright CL1500 imaging system. 6.Cell sorting. Cells were sorted using FACSAria II. 7.Behavioral data acquisition. Noldus EthoVision XT software v.17.0.
Data analysis	1.RNA-seq. RNA-seq reads was assessed using FastQC quality control tool. Reads were concatenated then trimmed using Trimmomatic. The RNA reads were aligned to the mouse mm10 reference genome using HISAT2. HISAT2-generated SAM files were sorted and converted into BAM files using Samtools. The sorted reads were assembled into transcripts using StringTie104. Next, StringTie-generated transcript lengths and abundance estimates were converted into count matrices using Tximport. Differential gene expression analysis was performed with false discovery rate (FDR)-adjusted P values using DESeq2. Data visualization was performed in R (version 4.0.3). 2.Flow cytometry. Data were analyzed with FlowJo software version 10. 3.Immunofluorescence. Images were processed using Fiji and LAS X. 4.RT-qPCR. Quantitative PCR data were analyzed by the delta-delta Ct method. 5.Western Blotting. Images were analyzed using Fiji. 6.Microbiota sequencing. QIIME2 was used to analyze rRNA 16S seq, including denoising and dereplicating with DADA2, taxonomy was assigned using the EZTaxon EZ-biocloud, release May 2018 formatted for QIIME. beta-diversity was assessed by PCoA plots and PERMANOVA based on weighted and unweighted UniFrac distances. Changes in relative abundance was determined by linear discriminant analysis effect size (LEfSe). 7. Statistical analysis. Statistical analysis was performed using GraphPad Prism 9 software.

For manuscripts utilizing custom algorithms or software that are central to the research but not yet described in published literature, software must be made available to editors and reviewers. We strongly encourage code deposition in a community repository (e.g. GitHub). See the Nature Portfolio guidelines for submitting code & software for further information.

Data

Policy information about availability of data

All manuscripts must include a data availability statement. This statement should provide the following information, where applicable:

- Accession codes, unique identifiers, or web links for publicly available datasets
- A description of any restrictions on data availability
- For clinical datasets or third party data, please ensure that the statement adheres to our policy

BioProject accession number PRJNA936187, [https://www.ncbi.nlm.nih.gov/sra/?term=PRJNA936187].

Human research participants

Policy information about studies involving human research participants and Sex and Gender in Research.

Reporting on sex and gender	n/a
Population characteristics	n/a
Recruitment	n/a
Ethics oversight	n/a

Note that full information on the approval of the study protocol must also be provided in the manuscript.

Field-specific reporting

Please select the one below that is the best fit for your research. If you are not sure, read the appropriate sections before making your selection.

☒ Life sciences ☐ Behavioural & social sciences ☐ Ecological, evolutionary & environmental sciences

For a reference copy of the document with all sections, see nature.com/documents/nr-reporting-summary-flat.pdf

Life sciences study design

All studies must disclose on these points even when the disclosure is negative.

Sample size	In our study, 3-10 mice were used per group and every experiment was reproduced at least twice, which is commonly used for in vivo studies to alleviate unnecessary animal suffering. This sample size was chosen to establish a power of greater or equal to 80% based on web tools for sample size and power calculation.
Data exclusions	Outliers (a data point that differs significantly from other observations) were excluded as determined by the following: Any value that is 1.5 x IQR greater than the third quartile is designated as an outlier and any value that is 1.5 x IQR less than the first quartile is also designated as an outlier. Outliers may have occurred due to a variability in the measurement, an indication of novel data, or it may be the result of experimental error.
Replication	All experiments were performed at least twice to assure rigor and reproductibility, and all attempts at replication were successful.
Randomization	Mice were randomly assigned to the experimental groups.
Blinding	For all experiments the investigator was blinded to the different groups.

Reporting for specific materials, systems and methods

We require information from authors about some types of materials, experimental systems and methods used in many studies. Here, indicate whether each material, system or method listed is relevant to your study. If you are not sure if a list item applies to your research, read the appropriate section before selecting a response.

Materials & experimental systems

n/a	Involved in the study
☐	☒ Antibodies
☐	☒ Eukaryotic cell lines
☒	☐ Palaeontology and archaeology
☐	☒ Animals and other organisms
☒	☐ Clinical data
☒	☐ Dual use research of concern

Methods

n/a	Involved in the study
☒	☐ ChIP-seq
☐	☒ Flow cytometry
☒	☐ MRI-based neuroimaging

Antibodies

Antibodies used

For flow cytometry: AF700 anti-CD45 (Biolegend, #103128, 1:300), BV785 anti-CD11b (BD Biosciences, #740861, 1:300), BV605 anti-CD3ε (Biolegend, #100351, 1:300), APC anti-TCRγδ (eBioscience, #17-5711-82, 1:300), FITC anti-Vγ1.1/Cr4 (Biolegend, clone 2.11, #141104, 1:300), PE anti-Vγ2 ("anti-Vγ4", Biolegend, clone UC3-10A6, #137706, 1:300), BV421 anti-IFN-γ (Biolegend, #505830, 1:300), PE/Cyanine7 anti-IL-17A (Biolegend, #506922, 1:300), BUV661 anti-CD45 (BD Biosciences, #565079, 1:1000), BB515 anti-CD11b (BD Biosciences, #564454, 1:1000), APC-Fire 750 anti-Ly-6C (Biolegend, #128046, 1:800), PE/Dazzle594 anti-CX3CR1 (Biolegend, #149013, 1:400), BB700 anti-CCR2 (BD Biosciences, #747965, 1:400 – stained separately at 37°C for 15 minutes), BV605 anti-I-A/I-E ("MHC class II", BD Biosciences, #563413, 1:800), BV650 anti-CD3ε (BD Biosciences, #564378, 1:200), BUV496 anti-CD4 (BD Biosciences, #612952, 1:400), BUV805 anti-CD8 (BD Biosciences, #564920, 1:400), FITC anti-FoxP3 (eBioscience, #11-5773-82, 1:200), PE anti-LAP (Biolegend, #141404, 1:100), PE/Dazzle 594 anti-IL10 (Biolegend, #505034, 1:100), Fixable Viability Dye eFluor 506 (eBioscience, #65-0866-18, 1:800). BUV563 anti-Ly-6G (BD Biosciences, #565707, 1:200).

For tissue staining: rabbit monoclonal anti-APP (Abcam, #ab32136, 1:4000, EDTA antigen retrieval), mouse monoclonal anti-ptau Ser202/Thr205 AT8 (eBioscience, #MN1020, 1:100, citrate antigen retrieval), rabbit anti-Iba1 polyclonal (Wako Chemicals, #019-19741, 1:500, EDTA antigen retrieval), rabbit anti-iNOS polyclonal (Abcam, #ab15323, 1:100, EDTA antigen retrieval), rabbit anti-GFAP polyclonal (Abcam, #ab72600, 1:3000, citrate antigen retrieval), rabbit polyclonal anti-beta III tubulin ("anti-Tuj1", Abcam, #ab18207, 1:5000, citrate antigen retrieval) and rabbit polyclonal anti-EAAT2 (Abcam, #ab41621, 1:5000, EDTA antigen retrieval). For immunofluorescence, the following fluorophores were used: Alexa Fluor 488 Tyramide (Life Technologies, #B40953), Alexa Fluor 555 Tyramide (Life Technologies, #B40955), Alexa Fluor 594 Tyramide (Life Technologies, #B40957) and Alexa Fluor 647 Tyramide (Life Technologies, #B40958). Nuclei were counterstained with NucBlue Fixed cell stain (Life Technologies, #R37606).

For sorting of microglial cells: APC/Cy7 anti-CD45 (Biolegend, #103116, 1:100), PE/Cy7 anti-CD11b (Biolegend, #101216, 1:100), FITC anti-Ly-6C (Biolegend, #128006, 1: 200) antibodies as well as the microglia specific 1:800 APC anti-4D4 antibody provided by Oleg Butovsky.

For Western blotting: primary antibodies used were: rabbit monoclonal anti-HMGB1 (Cell signaling, #6893S, 1:1000), rabbit polyclonal anti-ZO1 (Abcam, #ab96587, 1:1000), rabbit monoclonal anti-Occludin (Abcam, #ab167161, 1:2000) and rabbit monoclonal anti-GAPDH (Cell signaling, #5174T, 1:2000). Membranes were then washed three 3 times and incubated with the secondary antibody horseradish peroxidase (HRP)-linked goat anti-rabbit (Cell signaling, #7074P2, 1:2000) on a shaker for 1 hour. The membranes were washed again, incubated with Pierce ECL Plus substrate (eBioscience, # 32134) and exposed on iBright CL1500 imaging system (Thermo Fisher Scientific). Images were imported into Fiji and band intensity was quantified relative to GAPDH.

Gamma delta T cell depleting antibodies: anti-Vγ1 (Bio X Cell, clone 2.11, #BE0257), anti-Vγ2 ("anti-Vγ4", Bio X Cell, clone UC3-10A6, #BE0168) and anti-TCRγδ (Bio X Cell, clone UC7-13D5, #BE0070) monoclonal antibodies.

In vitro culture studies: 1 µg/mL of anti-CD3 (Biolegend, #100340) was added to all inserts. According to the experimental condition, 10 µg/mL of anti-TGF-β (Bio X Cell, clone 1D11.16.8, #BE0057) or anti-IL-17a (Bio X Cell, clone 17F3, #BE0173) were added to the inserts.

Validation

Antibody dilutions were used as recommended by the manufactures. All antibodies employed in this study are well characterized and broadly used by the research community. Validation of all primary antibodies for the species and applications can be found at the website of the company, for example, Biolegend, Abcam, and BD Biosciences where the antibodies were purchased. Microglia staining 4D4 antibody was developed in our laboratory and has been extensively used. Refs 61, 62. Krasemann, S. et al. The TREM2-APOE Pathway Drives the Transcriptional Phenotype of Dysfunctional Microglia in Neurodegenerative Diseases. Immunity 47, 566-581 e569 (2017). Butovsky, O. et al. Modulating inflammatory monocytes with a unique microRNA gene signature ameliorates murine ALS. J Clin Invest 122, 3063-3087 (2012).

Eukaryotic cell lines

Policy information about cell lines and Sex and Gender in Research

Cell line source(s)	HEK293T packaging cells were purchased from ATCC
Authentication	Authentication was provided by ATCC
Mycoplasma contamination	As per ATCC, the cell line was negative for mycoplasma contamination.
Commonly misidentified lines (See ICLAC register)	n/a

Animals and other research organisms

Policy information about studies involving animals; ARRIVE guidelines recommended for reporting animal research, and Sex and Gender in Research

Laboratory animals	Male and female mice were used in this study. C57BL/6 wild-type (WT) (#000664), TCRδ−/− (#002120), IFNγ−/− (#002287) and IL17af −/− (#034140) mice were purchased from the Jackson Laboratory. Animals were housed in a conventional specific pathogen-free facility at the Building for Transformative Medicine, Brigham and Women's Hospital, under standard 12-hour light/dark cycle conditions.
Wild animals	No wild animals were used in the study.
Reporting on sex	Both male and female mice were used to investigate sex dependency in our model.
Field-collected samples	No field collected samples were used in the study.
Ethics oversight	All experiments were reviewed and overseen by the institutional animal use and care committee at Brigham and Women's Hospital in accordance with NIH guidelines for the humane treatment of animals. IACUC Protocol number: 2016N000230.

Note that full information on the approval of the study protocol must also be provided in the manuscript.

Flow Cytometry

Plots

Confirm that:

☒ The axis labels state the marker and fluorochrome used (e.g. CD4-FITC).

☒ The axis scales are clearly visible. Include numbers along axes only for bottom left plot of group (a 'group' is an analysis of identical markers).

☒ All plots are contour plots with outliers or pseudocolor plots.

☒ A numerical value for number of cells or percentage (with statistics) is provided.

Methodology

Sample preparation

Generation of microglia single-cell suspension. Mice were intracardially perfused with ice cold Mg2+ and Ca2+ free 1X Hank's balanced salt solution (HBSS; Gibco, #14175). Brains were gently dissected from the overlying skull with the peri-contusion brain tissue quickly removed and placed on ice. Brains were mechanically homogenized using Dounce homogenizers in ice cold HBSS. Brains were homogenized 10 times each with the loose and tight pestles while simultaneously rotating the pestle. The cell suspension was then transferred to prechilled 15 mL tubes and passed through an HBSS pre-wet 70 µm cell strainer (Fisher Scientific, #22363548). Cell suspensions were then centrifuged at 1500 rpm for 5 minutes at 4 °C. Debris and myelin were removed using a modified room-temperature Percoll gradient82. Briefly, cell pellets were resuspended in 5 mL room-temperature 30% Percoll Plus cell separation liquid media (GE Healthcare Biosciences, #17-5445-01) diluted in HBSS and then spun for 20 minutes at 1800 rpm with acceleration rate of 5 and deceleration rate of 3. Microglia cells subsequently pellet at the bottom of the 15 mL tubes with myelin and debris floating above Percoll Plus solution. The cell pellet was washed with 10 mL ice cold FACS buffer (Mg2+ and Ca2+ free HBSS with 2% FBS, 0.4% EDTA 0.5 M and 2.5% HEPES 1 M) and spun again for 5 minutes at 1500 rpm at 4 °C. All samples were then resuspended in 100 µl of ice cold FACS buffer containing APC/Cy7 anti-CD45 (Biolegend, #103116, 1:100), PE/Cy7 anti-CD11b (Biolegend, #101216, 1:100), FITC anti-Ly-6C (Biolegend, #128006, 1:200) antibodies as well as the microglia specific 1:800 APC anti-4D4 antibody provided by Oleg Butovsky. Cells were stained for 20 minutes on ice. Samples were then washed in 10 mL ice cold FACS buffer and spun for 5 minutes at 1500 rpm and then resuspended in 200 µl of ice cold FACS buffer. 1,000 live CD45lowCD11bposLy-6Cneg4D4pos microglial cells were sorted on a BD FACSAria II using the 70 µm nozzle with a sort speed of approximately 10,000 events per second. Dead cells were excluded using 7-AAD viability staining solution (Biolegend, #420404, 1:100). Gating strategy for microglia sorting is shown in Extended Data Fig. 9a, b. The cells were sorted into prechilled 1.5 mL tubes containing 1% 2-mercaptoethanol in TCL buffer (Qiagen, #1031576). After sorting, the cells were immediately short-spun, placed on dry ice and stored in -80°C freezer.

Brain edema. Brains were removed at 72 hours after CCI and the ipsilateral hemisphere was weighed (wet weight). The hemisphere was then dried at 60 °C for 48 hours, and dry weights were obtained. The percentage of brain water content was expressed as (wet-dry weight)/wet weight × 100 as previously described106.

RNA sequencing. At the end of FACS sorting, single cell suspensions were plated in a prechilled 96-well twin.tec PCR plate LoBind, full skirted plate (Eppendorf, #0030129512) and shipped to the Broad Institute for Smart-seq2 RNA sequencing. Samples were processed for cDNA generation and Illumina Nextera XT library construction. Sequencing data was generated using 2x38 bp paired end sequencing on the NextSeq500. Transcript-level gene expression analysis of the raw RNA-seq reads was performed as previously described93. First, quality of the raw RNA-seq reads was assessed using FastQC quality control tool for high throughput sequence data. Reads were concatenated then trimmed using Trimmomatic. The RNA reads were aligned to the mouse mm10 reference genome using HISAT296. HISAT2-generated SAM files were sorted and converted into BAM files using Samtools. The sorted reads were assembled into transcripts using StringTie. Next, StringTie-generated transcript lengths and abundance estimates were converted into count matrices using Tximport. Differential gene expression analysis was performed with false discovery rate (FDR)-adjusted P values using DESeq2 with an adjusted P cutoff value of

0.05. Data visualization was performed in R (version 4.0.3). Heatmaps and clustering were generated using heatmap.2 from the gplots package. For clustering, the z-scores were calculated using the mean expression of biological replicates per disease stage/condition and then subsequently clustered using K-means. Two-dimensional principal component analysis (PCA) plots were generated using the ggplot2 package and prcomp from the stats package. MA plots were generated using ggmaplot from the ggpubr package. Pathway analysis was performed using gene set enrichment analysis (GSEA). GSEA or GSEA Preranked analyses were used to generate enrichment plots for RNA-seq data using MSigDB molecular signatures for canonical pathways: hallmark (h.all) and gene ontology (c5.cp.all) pathways. From the enrichplot package, enrichment plots were generated using gseaplot2, dot plots were generated using dotplot and gene networks were visualized using cnetplot. Statistical analysis in GSEA was determined by one-tailed t-test in GSEA. In all cases, FDR values were derived using the Benjamini-Hochberg test and P values were calculated. To identify regulators of gene expression networks, ingenuity pathway analysis (IPA) software (Qiagen) was used by inputting gene expression datasets. 'Canonical pathways' and 'upstream analysis' metrics were considered significant at $P < 0.05$. To identify regulatory networks, once a specific regulator was identified, the 'Build>Grow' function was used to identify molecules of the selected network. Statistical analysis using Qiagen IPA was carried out with a right-tailed Fisher's exact test.

Flow cytometry. Microglia were separated using Percoll Plus as described above and spleen, deep cervical lymph nodes (cLN) and blood were harvested before intracardiac perfusion. All tissues were taken from the same animal. cLN and spleens were macerated through a 70 µm cell strainer (Fisher Scientific, #22363548) and centrifuged at 300 g for 5 minutes at 4°C. For spleens, RBCs were lysed using ACK lysing buffer (eBioscience, #A1049201). Both cLN and spleen cells were washed in ice cold FACS buffer prior to staining. Blood was initially collected in Vacutainer heparinized tubes (BD Biosciences, #366664) then stained and lysed using FACS Lysing Solution according to manufacturer's instructions (BD Biosciences, #349202, 1:10). For intracellular cytokine staining, cells were stimulated for 4 hours with 50 ng/mL phorbol 12-myristate 13-acetate (PMA; Sigma-Aldrich, #P1585), 1 µM ionomycin (Sigma-Aldrich, #I3909) and 1 µg/mL GolgiStop protein-transport inhibitor containing monensin (BD Biosciences, #554724) diluted in Iscove's Modified Dulbecco's Medium (IMDM; Gibco, #31980030) containing heat-inactivated 10% fetal bovine serum (FBS; Gibco, #10438026), 100 U/mL penicillin-streptomycin mixture (Lonza, #DE17-602E), 55 µM 2-mercaptoethanol (Gibco, #21985023) and 1% non-essential amino acids (Lonza, #BE13-114E) prior to staining with antibodies. Fc receptors were blocked with anti-mouse CD16/CD32 (Bio X Cell, #BE0307, 1:50) for 15 minutes on ice. Surface markers were stained for 20 min at 4 °C in FACS buffer then fixed and permeabilized with Foxp3/Transcription Factor Staining Buffer Set (eBioscience, #00-5523-00). Cells were then stained for intracellular cytokines and washed in FACS buffer. Zombie Aqua Fixable Viability Kit (Biolegend, #423102, 1:1000) was used to exclude dead cells. Flow cytometric acquisition was performed on LSRFortessa and FACSSymphony A5 (BD Biosciences) using DIVA software (BD Biosciences) and data were analyzed with FlowJo software version 10 (TreeStar Inc.). The staining antibodies used are AF700 anti-CD45 (Biolegend, #103128, 1:300), BV785 anti-CD11b (BD Biosciences, #740861, 1:300), BV605 anti-CD3ε (Biolegend, #100351, 1:300), APC anti-TCRγδ (eBioscience, #17-5711-82, 1:300), FITC anti-Vγ1.1/Cr4 (Biolegend, clone 2.11, #141104, 1:300), PE anti-Vγ2 ("anti-Vγ4", Biolegend, clone UC3-10A6, #137706, 1:300), BV421 anti-IFN-γ (Biolegend, #505830, 1:300), PE/Cyanine7 anti-IL-17A (Biolegend, #506922, 1:300), BUV661 anti-CD45 (BD Biosciences, #565079, 1:1000), BB515 anti-CD11b (BD Biosciences, #564454, 1:1000), APC-Fire 750 anti-Ly-6C (Biolegend, #128046, 1:800), BUV563 anti-Ly-6G (BD Biosciences, #565707, 1:200), PE/Dazzle594 anti-CX3CR1 (Biolegend, #149013, 1:400), BB700 anti-CCR2 (BD Biosciences, #747965, 1:400 – stained separately at 37°C for 15 minutes), BV605 anti-I-A/I-E ("MHC class II", BD Biosciences, #563413, 1:800), BV650 anti-CD3ε (BD Biosciences, #564378, 1:200), BUV496 anti-CD4 (BD Biosciences, #612952, 1:400), BUV805 anti-CD8 (BD Biosciences, #564920, 1:400), FITC anti-FoxP3 (eBioscience, #11-5773-82, 1:200), PE anti-LAP (Biolegend, #141404, 1:100), PE/Dazzle 594 anti-IL10 (Biolegend, #505034, 1:100), Fixable Viability Dye eFluor 506 (eBioscience, #65-0866-18, 1:800).

Immunostaining. Mice were intracardially perfused with 10 mL ice cold 1X phosphate buffered saline (PBS) followed by 10 mL ice cold 4% paraformaldehyde (PFA). Brains were gently dissected, post-fixed in 4% PFA overnight at 4°C and dehydrated in 30% sucrose for 3 days at 4°C. Brains were then paraffin embedded and 5-µm sections were obtained by a microtome and stored at room temperature. All immunostaining was performed on the Leica Bond III automated staining platform using the Leica Biosystems Refine Detection Kit (Leica Biosystems, #DS9800).

Quantitative PCR. Freshly dissected brain tissue was stored in RNAlater (Sigma-Aldrich, #R0901) at 4°C and processed the following day. Brains were then electrically homogenized (Kinematica, #PT1200E) in buffer RLT (Qiagen, #79216) containing 1% 2-mercaptoethanol. Lysate was centrifuged for 3 minutes at maximum speed at 4°C. Lysate was transferred to a genomic DNA eliminator column and RNA extraction was performed as per manufacturer's instructions (RNeasy Plus Mini Kit, Qiagen, #74134). For RNA extraction of freshly FACS-sorted cells, RNeasy Plus Micro Kit, (Qiagen, #74034) was used. Next, RNA was reverse-transcribed using the High-Capacity cDNA Reverse Transcription Kit with RNase Inhibitor (Life Technologies, #4374966). Quantitative real-time PCR was then performed on the cDNA using TaqMan Fast Universal PCR Master Mix (2X) no AmpErase UNG (Life Technologies, #4352046) with a Vii 7 real-time PCR system (Applied Biosystems) with the following primers and probes: Il1b (Mm00434228_m1), Il6 (Mm00446190_m1), Il10 (Mm01288386_m1), Il12a (Mm00434169_m1), Il13 (Mm00434204_m1), Il17a (Mm00439618_m1), Il18 (Mm00434226_m1), Il23a (Mm00518984_m1), Il33 (Mm00505403_m1), Ifng (Mm01168134_m1), Tnf (Mm00443258_m1), Tgfb1 (Mm01178820_m1), Foxp3 (Mm00475162_m1), Ccl2 (Mm00441242_m1), Ccl5 (Mm01302427_m1), Ccl20 (Mm01268754_m1), Cxcl10 (Mm00445235_m1), Gata3 (Mm00484683_m1), Ccr4 (Mm01963217_u1), Ccr5 (Mm01963251_s1), Ccr6 (Mm99999114_s1) and Ifngr1 (Mm00599890_m1) in addition to in-house-designed (and synthesized by Life Technologies) probes targeting Vγ1 (AI89KL3) and Vγ4 (AIBJXX4) chain segments. Quantitative PCR data were analyzed by the delta-delta Ct method by normalizing the expression of each gene to Gapdh (Mm99999915_g1).

Western blotting. Freshly dissected brain tissue was electrically homogenized (Kinematica, #PT1200E) in RIPA lysis and extraction buffer (eBioscience, #89900) containing 1% Halt protease and phosphatase inhibitor cocktail (eBioscience, #78441). Samples were kept on ice for 5 minutes and protein lysates were then obtained by centrifugation at maximum speed for 10 minutes at 4°C. Protein quantification was performed by Nanodrop (eBioscience). Equal amount of total protein was obtained by dilution into 4X Bolt LDS Sample Buffer (Invitrogen, #B0007), 10X Bolt Sample Reducing Agent (Invitrogen, #B0009) and deionized water up to a total volume of 40 µl. Lysates were boiled at 70°C for 10 minutes. Next, samples along with a SeeBlue Plus2 pre-stained protein standard (Invitrogen, #LC5925) were loaded into Bolt 4-12% Bis-Tris mini protein gels (Invitrogen, #NW04125BOX) and SDS-PAGE was performed at 120 V in diluted 20X Bolt MES SDS running buffer (Invitrogen, #B0002). Proteins were blotted to the transfer membrane using the iBlot dry blotting system (Invitrogen, #IB1001) and nitrocellulose mini iBlot Transfer Stack (Invitrogen, #IB301002). Membranes were then cut horizontally

according to the molecular weights of the target proteins and guided by the protein standard ladder. Membranes were blocked for 30 minutes on a shaker using StartingBlock T20 tris-buffered saline (TBS) blocking buffer (eBioscience, #37543) that already contains 0.05% Tween-20. Membranes were then washed 3 times with the blocking buffer. Primary antibodies were diluted in blocking buffer and incubated with the membranes overnight at 4 °C.

Depletion of γδ T cell subsets. WT mice were depleted of their Vγ1 cell, Vγ4 cells as well as total TCRγδ cells using 200 µg anti-Vγ1 (Bio X Cell, clone 2.11, #BE0257), 200 µg anti-Vγ2 (“anti-Vγ4”, Bio X Cell, clone UC3-10A6, #BE0168) and 200 µg anti-TCRγδ (Bio X Cell, clone UC7-13D5, #BE0070) monoclonal antibodies, respectively. The monoclonal antibodies were injected intraperitoneally 24 hours before TBI.

Purification and adoptive transfer of γδ T cell subsets. Splenocytes were isolated from C57BL/6J WT mice as described above. γδ T cells were first purified and enriched using TCRγδ T cell isolation microbeads kit (Miltenyi Biotech, #130-092-125) on a magnetic MACS separator prior to sorting. Eluted cells were pooled and stained in FACS buffer containing APC/Cy7 anti-CD45 (Biolegend, #103116, 1:100), PE/Cy7 anti-CD3ε (Biolegend, #152313, 1:100), APC anti-TCRγδ (eBioscience, #17-5711-82, 1:300), FITC anti-Vγ1.1/Cr4 (Biolegend, clone 2.11, #141104, 1:300) and PE anti-Vγ2 (“anti-Vγ4”, Biolegend, clone UC3-10A6, #137706, 1:300) for 20 minutes on ice. Dead cells were excluded using 7-AAD viability staining solution (Biolegend, #420404, 1:100). CD45posCD11bnegCD3εposTCRγδposVγ1posVγ4neg and CD45posCD11bnegCD3εposTCRγδposVγ1negVγ4pos subsets were sorted using FACSAria II (BD Bioscience). Gating strategy for cell sorting is as shown in Extended Data Fig. 9c. TCRγδ cell subsets were sorted and immediately transferred into TCRδ-/- mice. Each mouse received 5×105 cells intravenously at the same time of TBI.

Transwell culture. Microglia and γδ T cell subsets were isolated as described above. Microglia were directly sorted into a microglia culture media composed of 10% fetal bovine serum (FBS; Gibco, #10438026), 100 U/mL penicillin-streptomycin mixture (Lonza, #DE17-602E), 55 µM 2-mercaptoethanol (Gibco, #21985023), 1% non-essential amino acids (Lonza, #BE13-114E) supplemented in Dulbecco's Modified Eagle Medium (DMEM)/F-12 Glutamax media (Gibco, #10565018). γδ T cells were directly sorted into a lymphocyte culture media composed of 10% fetal bovine serum (FBS; Gibco, #10438026), 100 U/mL penicillin-streptomycin mixture (Lonza, #DE17-602E), 55 µM 2-mercaptoethanol (Gibco, #21985023), 1% sodium pyruvate (Lonza, #BE13-115E) and 1% HEPES (Lonza, #BE17-737E) supplemented in Roswell Park Memorial Institute (RPMI) 1640 media (Gibco, #11875119). Cell count was performed and 100,000 microglia cells per condition were plated in the lower chambers of 24-well plates (Corning, #3524) and 50,000 γδ T cells per condition were plated in 0.4 µm inserts (Millipore, #MCHT24H48). 1 µg/mL of anti-CD3 (Biolegend, #100340) was added to all inserts. According to the experimental condition, 10 µg/mL of anti-TGF-β (Bio X Cell, clone 1D11.16.8, #BE0057) or anti-IL-17a (Bio X Cell, clone 17F3, #BE0173) were added to the inserts. Cells were co-cultured at 37 °C in a humidified incubator with 5% CO2 for 48 hours after which microglia was taken for RNAseq.

Lentivirus generation and injection. Third-generation lentivirus packaging was provided by AMSBIO (Massachusetts, USA). Briefly, a transfer plasmid carrying the gene of interest “Ifngr1” was co-transfected with a proprietary envelope plasmid encoding VSV-G and packaging plasmids encoding Gag/Pol and Rev into HEK 293T packaging cells. Cd11b was used as a promoter and emGFP was inserted to act as a fluorescent protein. After 48 hours of incubation, the supernatant is collected and centrifuged to remove cell debris and then filtered. Lentiviral particles were subsequently concentrated with polyethylene glycol. Lentivirus titer was measured using the p24 ELISA method. Briefly, HIV-1 p24 antigen was captured by anti-p24 coated microtiter wells and sandwiched with biotinylated secondary anti-p24 antibody. Subsequently, a streptavidin-HRP conjugate and a substrate were added. Color intensity was measured spectrophotometrically to indicate the level of p24 in the sample, which was then quantified against a p24 standard curve. p24 value was then correlated to virus titer. The pellet containing lentivirus was resuspended in PBS and aliquots were stored at −80 °C. Quality control steps included titer measurement, sterility testing for bacteria and fungi, mycoplasma detection, fluorescent protein transduction test and drug selection test. The vector without insertion of mi-shIFN-γR1 was used as control.
Mice were injected with approximately 1 × 107 IU in the bilateral ventricles using a 25-µl Hamilton syringe (Sigma-Aldrich, #20787) on a stereotaxic alignment system at the following coordinates: 1.0 mm caudal to bregma, 2.0 mm laterally and 2.5 mm below the skull surface. Injection speed was maintained at 1 µl/min to prevent leaking.

Behavioral testing. All behavioral experiments were carried out during daylight hours in a blinded fashion. Mice were allowed to acclimate for 3-4 weeks prior to testing. At the day of testing, animals were acclimatized to the behavioral rooms for at least 30 minutes. Animals from different groups were tested consecutively. For the depletion and adoptive transfer experiments, mice underwent all behavioral assays beginning with the rotarod test, followed by the open field test and then the Morris water maze test. To account for social interactions, sham and TBI mice of the same genetic background were mixed in the same cage. With the exception of rotarod and marble burying tests, data were digitally recorded using a camera-enabled Noldus EthoVision XT software.

Instrument	All instruments used are described in each section above.
Software	All softwares used are described in each section above.
Cell population abundance	For sorting, cell were purified by sorting on an BD Aria III instrument.
Gating strategy	Gating strategy for microglial sorting is shown in Extended Data Figure 9.

☒ Tick this box to confirm that a figure exemplifying the gating strategy is provided in the Supplementary Information.
